# A multicenter performance evaluation of aztreonam-avibactam gradient diffusion susceptibility testing for *Enterobacterales*

**DOI:** 10.1128/jcm.01749-25

**Published:** 2026-04-20

**Authors:** Tina I. Bui, Edith Csiki-Fejer, Sarah Prudhomme, Louise Bossy, Dwight J. Hardy, Mandy Wootton, Gilles Zambardi, Melanie L. Yarbrough

**Affiliations:** 1 Department of Pathology and Immunology, Washington University School of Medicine 12275 , Saint Louis, Missouri, USA; 2 bioMérieux Clinical Affairs, Saint Louis, Missouri, USA; 3 bioMérieux Clinical Affairs, Marcy-l’Etoile, France; 4 Department of Microbiology and Immunology, University of Rochester Medical Center 548028, Rochester, New York, USA; 5 Public Health Wales Microbiology, University Hospital of Wales 97609, Cardiff, United Kingdom; Endeavor Health, Evanston, Illinois, USA

**Keywords:** aztreonam-avibactam, *Enterobacterales*, gradient diffusion, ETEST AZA

## Abstract

Aztreonam-avibactam (AZA) is a new monobactam/beta-lactamase inhibitor combination that recently received regulatory approval in the United States (U.S.) and Europe. In this study, we evaluated the performance of ETEST AZA, a gradient diffusion method, compared to reference broth microdilution for 650 challenge (*n* = 77) and clinical (*n* = 573) *Enterobacterales* isolates across four study sites. Using the U.S. Food and Drug Administration (FDA) interpretive breakpoints for ETEST AZA, challenge isolates demonstrated a 97.3% (72/74) essential agreement (EA), 93.2% (69/74) categorical agreement (CA), 0% very major errors (VME) and major errors (ME), and 6.8% (5/74) minor errors (mE). Clinical isolates demonstrated a 95.5% (504/528) EA, 98.7% (521/528) CA, 0% VME, 0.2% (1/516) ME, and 1.1% (6/528) mE. Overall, the combined set achieved a 95.7% (576/602) EA and 98.0% (590/602) CA, with 0% VME, 0.2% (1/577) ME, and 1.8% (11/602) mE. Using the European Committee on Antimicrobial Susceptibility Testing (EUCAST) interpretive breakpoints, challenge isolates demonstrated a 96.1% (74/77) EA, 93.5% (72/77) CA, and 31.3% (5/16) VMEs, while clinical isolates demonstrated a 94.1% (539/573) EA and 99.7% (571/573) CA including 7.7% (1/13) VME and 0.2% (1/560) ME. The combined set demonstrated a 94.3% (613/650) EA, 98.9% (643/650) CA, 0.2% (1/573) MEs, but 20.7% (6/21) VMEs due to the lack of an intermediate breakpoint that is addressed as a limitation in the European package insert. Both bias and trending were in alignment with regulatory requirements. In reproducibility testing of 10 on-scale strains, 100% (270/270) of the results were within ±1 doubling dilution of the modal minimal inhibitory concentration. In conclusion, ETEST AZA provides accurate, reproducible susceptibility results that meet regulatory requirements for many *Enterobacterales* species when interpreted using FDA or EUCAST criteria.

**IMPORTANCE:**

Aztreonam-avibactam (AZA) is a novel monobactam/β-lactamase inhibitor combination approved for clinical use in the United States and Europe in 2025 and 2024, respectively. It is indicated for the treatment of multidrug-resistant Gram-negative bacteria that produce multiple classes of β-lactamases. Until recently, antimicrobial susceptibility testing for this combined agent relied on broth disk elution or microdilution using ceftazidime–avibactam and aztreonam combined. In this study, we evaluated the performance of the bioMérieux ETEST AZA gradient diffusion strip as a more streamlined method for clinical microbiology laboratories. Testing of 650 *Enterobacterales* isolates demonstrated that ETEST AZA met regulatory performance criteria using interpretive breakpoints from both the FDA and the European Committee on Antimicrobial Susceptibility Testing. Overall, our findings indicate that the ETEST AZA demonstrates acceptable performance for many *Enterobacterales* isolates and represents a practical alternative for implementing AZA susceptibility testing in clinical microbiology laboratories.

## INTRODUCTION

The 2024 Infectious Diseases Society of America (IDSA) guidance currently recommends combination therapy using ceftazidime-avibactam with aztreonam for serious, non–urinary tract infections caused by metallo-β-lactamase (MBL)–producing *Enterobacterales* (1). This regimen is relevant for organisms that harbor MBLs, most commonly *bla*_NDM_, and other serine β-lactamases, such as extended-spectrum β-lactamases (ESBLs), *Klebsiella pneumoniae* carbapenemases (KPCs), OXA-48-like enzymes, and AmpC β-lactamases. Aztreonam, a monobactam, is intrinsically stable to MBL-mediated hydrolysis, while avibactam, a non–β-lactam β-lactamase inhibitor, effectively inhibits serine β-lactamases but lacks activity against MBLs (2). When combined, avibactam prevents the degradation of aztreonam by serine β-lactamase–mediated hydrolysis, allowing aztreonam to remain effective against MBLs. Aztreonam-avibactam (AZA) as a single agent offers a streamlined therapeutic option compared to the combination of ceftazidime-avibactam and aztreonam, ensuring consistent simultaneous delivery of both active components. This dual mechanism positions AZA as a promising treatment alternative for infections caused by multidrug-resistant organisms that co-express MBLs and serine β-lactamases, for which there are limited treatment options (3).

Until recently, there was no approved β-lactam/β-lactamase inhibitor with activity against MBL-producing *Enterobacterales*. AZA is a novel monobactam/β-lactamase inhibitor combination approved by the European Commission in April 2024 for the treatment of complicated intra-abdominal infections (cIAI), hospital-acquired pneumonia (HAP), and complicated urinary tract infections (cUTI). In February 2025, the U.S. Food and Drug Administration (FDA) approved AZA for use in combination with metronidazole for treating cIAI caused by *Enterobacterales*. Effective clinical use of new antimicrobial agents depends on clinical microbiology laboratories’ ability to perform accurate susceptibility testing. Currently, few methods are available for AZA susceptibility testing. The Clinical and Laboratory Standards Institute (CLSI) endorses a broth disk elution (using ceftazidime-avibactam and aztreonam disks) and broth microdilution methods (4, 5), but this can be labor-intensive, limiting its feasibility in most clinical microbiology laboratories. Gradient diffusion susceptibility testing, such as the ETEST, provides a practical alternative. In this study, we conducted a multicenter performance evaluation of the FDA-cleared and CE-marked ETEST AZA (bioMérieux; La Balme Les Grottes, France) compared to the reference broth microdilution method.

## MATERIALS AND METHODS

### Study design

Prior to study initiation, each participating site obtained approval or a waiver from its respective institutional review board. A total of 650 isolates were tested using the ETEST AZA and compared to the CLSI reference broth microdilution (BMD) method. Challenge isolate (*n* = 77) testing was performed at bioMérieux Clinical Affairs in St. Louis, MO. Clinical isolates (*n* = 573) included a combination of contemporary and archived *Enterobacterales*. Of the 650 total isolates, 52% (339/650) were stock isolates, and 48% (311/650) were contemporary isolates recovered from clinical specimens within 6 months of AZA testing. All clinical isolates tested were non-susceptible to a carbapenem and/or a third-generation cephalosporin based on interpretive breakpoints from CLSI M100, 33^rd^ edition (6) and EUCAST breakpoint tables version 13. Overall, 56% (319/573) of clinical isolates were nonsusceptible to aztreonam, of which 72% (230/319) were also nonsusceptible to carbapenems, and 80% (255/319) were nonsusceptible to third-generation cephalosporins. Testing of clinical and reproducibility isolates was conducted at the University Hospital of Wales (Wales, UK), the University of Rochester Medical Center (Rochester, NY), and Washington University School of Medicine (St. Louis, MO). The results from this clinical trial were used for FDA 510(k) clearance (6).

### ETEST AZA susceptibility testing

Gradient diffusion testing with ETEST AZA (bioMérieux, La Balme Les Grottes, France) was performed in accordance with the manufacturer’s instructions. The strip is impregnated with a continuous concentration gradient of aztreonam ranging from 0.016 to 256 µg/mL, combined with a fixed avibactam concentration of 4 µg/mL. For each isolate, a 0.5 McFarland bacterial suspension (1.0 McFarland for mucoid strains) was prepared in 0.85% saline by visual comparison to a standard or using an FDA-approved nephelometric device. Standardized bacterial suspensions were inoculated onto Mueller-Hinton agar plates (Becton Dickinson; Franklin Lakes, NJ) using sterile cotton swabs. ETEST AZA gradient diffusion strips were applied with sterile forceps. Minimal inhibitory concentrations (MICs) were determined following incubation aerobically at 35°C ± 2°C for 16–20 h. All testing sites received the ETEST reading guide and preliminary package insert, which included standardized instructions for interpretation, including guidance on reading hazes (considered growth except for *Proteus* swarming) and colonies within 3 mm of the gradient strip (considered true growth).

### Reference broth microdilution as a comparator method

Broth microdilution (BMD) testing was performed in parallel with gradient diffusion using the same McFarland suspension, following CLSI M07 and M100 guidelines (7, 8). BMD was conducted using 96-well microtiter plates prepared by bioMérieux (La Balme Les Grottes, France), with wells containing 0.1 mL of aztreonam in 2-fold serial dilutions ranging from 0.16 to 256 µg/mL, combined with a fixed avibactam concentration of 4 µg/mL in cation-adjusted Mueller-Hinton broth (Beckton Dickinson; Franklin Lakes, NJ). BMD microtiter plates were thawed at room temperature for 30–60 min and were inoculated and incubated within 4 h of thawing. The 0.5 McFarland suspension was diluted 1:100 in cation-adjusted Mueller-Hinton broth (Becton Dickinson; Franklin Lakes, NJ) for inoculation. Each well, except for the sterility control, was inoculated with 50 µL of the diluted bacterial suspension using a repeater or multichannel pipette. A purity check was performed by transferring 10 µL from the growth control wells and plating them onto blood agar using the four-quadrant streak method. Inoculum density checks were conducted for quality control strains, reproducibility isolates, and 10% of fresh clinical isolates. For this, 100 µL of the diluted bacterial suspension in Mueller-Hinton broth was added to 10 mL of 0.85% saline (1:1,000 dilution) and 100 µL was spread onto a blood agar plate using L-shaped spreaders. Colony counts were recorded after incubation aerobically at 35°C ± 2°C for 18–24 h, with an expected range of 20–80 colonies.

### Quality control

Quality control (QC) testing was conducted in accordance with CLSI-defined ranges for designated QC strains (8). Two QC organisms for ETEST AZA and three QC organisms for the reference method (BMD) were tested at each study site daily, for a total of at least 20 replicates at each study site: *Escherichia coli* ATCC 25922 (expected range for BMD and ETEST: 0.03/4–0.12/4 µg/mL), *Klebsiella pneumoniae* ATCC 700603 (expected range for BMD and ETEST: 0.06/4–0.5/4 µg/mL), and *Pseudomonas aeruginosa* ATCC 27853 (expected range for BMD: 2/4–8/4 µg/mL).

### Reproducibility testing

Reproducibility testing was conducted on 10 on-scale strains, defined as isolates with expected MICs within the quantifiable range of the ETEST AZA using the BMD reference method, as curated by bioMérieux Research and Development (Marcy-l’Etoile, France) and sent to clinical study sites for testing. All strains were tested exclusively using ETEST AZA across the three external clinical study sites, in triplicate over 3 different days to yield a total of 270 data points. Inoculum density checks were performed for all isolates to ensure consistency and accuracy of test conditions as previously described.

### Clinical and challenge isolates testing

A total of 573 clinical and 77 challenge *Enterobacterales* isolates (*N* = 650) were evaluated for susceptibility using both ETEST AZA and reference BMD performed concurrently as previously described. All challenge isolates were from bioMérieux’s internal collection, and testing was performed at bioMérieux Clinical Affairs. Clinical isolates were obtained from routine clinical specimens submitted to clinical microbiology laboratories as part of the standard of care. Clinical isolate testing was carried out at the three study sites: University of Rochester Medical Center (*n* = 193), Washington University School of Medicine (*n* = 190), and University Hospital of Wales in Cardiff (*n* = 190). Isolates were identified and/or reidentified to the species level using matrix-assisted laser desorption ionization–time of flight mass spectrometry (MALDI-TOF MS) at Washington University in St. Louis and University of Rochester using Bruker MALDI Biotyper (claim 4) and Vitek MS Prime (Knowledge Base V3.3) systems, or by whole genome sequencing on a NextSeq instrument at Cardiff. Of the 573 clinical isolates, 311 (54.3%) were contemporary isolates (isolated from standard of care within 6 months) and 262 (45.7%) were categorized as stock isolates (frozen isolates from standard of care with no time constraints on time from collection). All isolates were subcultured onto blood agar prior to testing. Frozen isolates underwent two subcultures on blood agar prior to susceptibility testing.

### Interpretation of results and data analysis

All clinical and challenge isolates tested were included in the performance analysis. Essential agreement (EA) was calculated as the proportion of isolates for which MIC values obtained by ETEST were within ±1 doubling dilution of those from the reference method. Categorical agreement (CA) was defined as the percentage of isolates with matching interpretive categories (susceptible, intermediate, or resistant) between ETEST and BMD, based on the EUCAST and FDA interpretive criteria in Table 1 (9, 10). Very major errors (VMEs) occurred when an isolate was classified as resistant by BMD but susceptible by ETEST. Major errors (MEs) were defined as isolates classified as susceptible by BMD but resistant by ETEST. Minor errors (mEs) included any discrepancies in which one method yielded a susceptible or resistant interpretation, while the other yielded an intermediate. Reproducibility rates were calculated as the percentage of the total number of results within ±1 doubling dilution of the modal MIC divided by the total number of tests performed. Trending was calculated based on the difference in percents of on-scale MICs one or more doubling dilutions lower or higher compared to the reference method (11). Bias was calculated per International Standards Organization (ISO) regulatory requirements as the difference in percentage of all ETEST MIC values that were higher or lower than the BMD reference values (12). A positive bias indicates that ETEST MICs tended to be higher than those obtained by BMD, while a negative bias indicates that ETEST MICs were lower.

**TABLE 1:** FDA and EUCAST interpretive criteria for aztreonam-avibactam MICs in *Enterobacterales^e^*

Organism	FDA MIC breakpoints^*a*^ (µg/mL)			EUCAST MIC breakpoints^*b*^ (µg/mL)		
	S	I	R	S^*c*^	I^*d*^	R
*Enterobacterales*	≤4/4	8/4	≥16/4	≤4/4	–^*f*^	>4/4

^*a*^ FDA breakpoints are available at www.fda.gov/STIC.

^*b*^ EUCAST AZA breakpoints are as reported in reference (8).

^*c*^ Susceptible, standard dosing regimen.

^*d*^ Susceptible, increased exposure.

^*e*^ MIC, minimal inhibitory concentration; S, susceptible; I, intermediate; R, resistant.

^*f*^ –, not defined.

## RESULTS

### Quality control and reproducibility of ETEST AZA

For BMD, the QC pass rates were 98.8% (83/84) for *E. coli* ATCC 25922, 100% (84/84) for *K. pneumoniae* ATCC 700603, and 98.8% (84/85) for *P. aeruginosa* ATCC 27853. The two out-of-range QC results on BMD for *E. coli* and *P. aeruginosa* were one doubling dilution above the expected range. QC duplicates from the same day for these strains were all within the expected range. Thus, these results were acceptable with an acceptance rate higher than 95%. For ETEST AZA, both *E. coli* ATCC 25922 and K. *pneumoniae* ATCC 700603 had QC pass rates of 100% (84/84). The QC strain *P. aeruginosa* ATCC 27853 was not tested on ETEST AZA as *P. aeruginosa* species is not a claimed species.

For reproducibility testing, 10 on-scale strains were tested for a total of 270 tests (90/site), including *E. coli, K. pneumoniae* (*n* = 2), *E. cloacae* complex (*n* = 2), *Citrobacter freundii* complex, *Klebsiella oxytoca*, *Serratia marcescens*, and *Citrobacter koseri* (*n* = 2). The modal MIC of each strain tested and the doubling dilution from this modal MIC across all sites is shown in Table 2. All strains tested within the expected range, demonstrating 100% reproducibility rate for the best-case (270/270) and at 98.5% (266/270) for the worst-case across the three study sites. The best-case calculation is based upon the assumption that the off-scale values are ±1 doubling dilution of the test mode, while off-scale values are considered more than one doubling dilution from the mode for the worst-case calculation. The four instances in which *K. pneumoniae* strain B were off-scale at ≤0.016 µg/mL were accepted in the best case calculation because results were within ±1 doubling dilution of the test mode at 0.016 µg/mL.

**TABLE 2:** Reproducibility of ETEST AZA^*a*^

Microorganism	Modal MIC (µg/mL)	No. of tests with doubling dilution from the modal MIC						
		Off scale	−2	−1	0	+1	+2	Off scale
*Escherichia coli*	16	0	0	13	14	0	0	0
*Klebsiella pneumoniae*								
Strain A	0.5	0	0	2	23	2	0	0
Strain B	0.016	4	0	0	21	2	0	0
*Enterobacter cloacae* complex								
Strain A	1	0	0	5	22	0	0	0
Strain B	2	0	0		26	1	0	0
*Citrobacter freundii* complex	1	0	0	0	18	9	0	0
*Klebsiella oxytoca*	0.25	0	0	4	23	0	0	0
*Serratia marcescens*	0.064	0	0	0	16	11	0	0
*Citrobacter koseri*								
Strain A	0.032	0	0	0	26	1	0	0
Strain B	0.5	0	0	0	27	0	0	0
**Total**		**4**	**0**	**24**	**216**	**26**	**0**	**0**

^*a*^ MIC, minimal inhibitory concentration; AZA, aztreonam-avibactam.

### Performance of ETEST AZA

A comparative analysis was conducted on 573 clinical isolates and 77 challenge isolates individually, as well as on the combined data set, using both FDA and EUCAST interpretive criteria in a full-scale analysis. The final performance of ETEST AZA for FDA interpretive criteria was determined based on 74 challenge and 528 clinical isolates, for a total of 602 isolates (Table 3). Forty-eight *K. oxytoca* isolates were not included for FDA performance analysis, as the EA for this species was below the acceptance criteria and is not included in the FDA (510k)-cleared package insert (13). Based on FDA breakpoints, 9.5% (7/74) *Enterobacterales* challenge and 0.9% (5/528) clinical isolates were resistant to AZA by BMD. ETEST AZA demonstrated 97.3% (72/74) EA and 93.2% (69/74) CA for challenge isolates, with 0% VME and ME and 6.8% (5/74) mE, all of which occurred in *E. coli*. Clinical isolates demonstrated 95.5% (504/528) EA and 98.7% (521/528) CA. Seven total errors were found, including one ME in *K. pneumoniae* and a total of six mE in *E. coli*, *E. cloacae* complex, and *S. marcescens*. When challenge and clinical isolates were combined, ETEST AZA achieved an overall EA of 95.7% EA (576/602) and 98.0% (590/602) CA. Although not included in the overall performance analysis using FDA interpretative criteria, EA for *K. oxytoca* was demonstrated to be 66.7% (2/3) for challenge isolates and 77.8% (35/45) for clinical isolates, with 100% CA for all isolates tested. Excluding results for *K. oxytoca* from EA, all ETEST AZA MIC discrepancies occurred on the lower end of the gradient diffusion strip, ranging from 0.064 to 0.25 µg/mL.

**TABLE 3:** Performance of ETEST AZA on clinical (*N* = 573) and challenge (*n* = 77) isolates^*b*^

Organism	FDA interpretative criteria									EUCAST interpretative criteria						
	No. of isolates	BMD results			ETEST AZA performance					No. of isolates	BMD results		ETEST AZA performance			
		S	I	R	EA	CA	VME	ME	mE		S	R	EA	CA	VME	ME
Challenge isolates																
*Escherichia coli*	30	17	8	5	29 (96.7)	25 (83.3)	0	0	5	30	17	13	29 (96.7)	25 (83.3)	5	0
*Klebsiella pneumoniae*	26	24	0	2	25 (96.2)	26 (100)	0	0	0	26	24	2	25 (96.2)	26 (100)	0	0
*Enterobacter cloacae* complex	7	6	1	0	7 (100)	7 (100)	0	0	0	7	6	1	7 (100)	7 (100)	0	0
*Citrobacter freundii* complex	5	5	0	0	5 (100)	5 (100)	0	0	0	5	5	0	5 (100)	5 (100)	0	0
*Klebsiella oxytoca^a^*	–^*c*^	–	–	–	–	–	–	–	–	3	3	0	2 (66.7)	3 (100)	0	0
*Serratia marcescens*	2	2	0	0	2 (100)	2 (100)	0	0	0	2	2	0	2 (100)	2 (100)	0	0
*Klebsiella aerogenes*	2	2	0	0	2 (100)	2 (100)	0	0	0	2	2	0	2 (100)	2 (100)	0	0
*Proteus mirabilis*	2	2	0	0	2 (100)	2 (100)	0	0	0	2	2	0	2 (100)	2 (100)	0	0
**Challenge Total (%**)	**74**	**61** (**82.4**)	**9** (**12.2**)	**7** (**9.5**)	**72** (**97.3**)	**69** (**93.2**)	**0** (**0**)	**0** (**0**)	**5** (**6.8**)	**77**	**61** (**79.2**)	**16** (**20.8**)	**74** (**96.1**)	**72** (**93.5**)	**5** (**31.3**)	**0** (**0**)
Clinical isolates																
*Escherichia coli*	120	111	6	3	116 (96.7)	116 (96.7)	0	0	4	120	111	9	116 (96.7)	118	1	1
*Klebsiella pneumoniae*	92	92	0	0	87 (94.6)	91 (98.9)	0	1	0	92	92	0	87 (94.6)	92	0	0
*Enterobacter cloacae* complex	60	58	1	1	54 (90.0)	59 (98.3)	0	0	1	60	58	2	54 (90.0)	60	0	0
*Citrobacter freundii* complex	45	45	0	0	43 (95.6)	45 (100)	0	0	0	45	45	0	43 (95.6)	45	0	0
*Klebsiella oxytoca^a^*	–	–	–	–	–	–	–	–	–	45	44	1	35 (77.8)	45	0	0
*Serratia marcescens*	31	30	0	1	30 (96.8)	30 (100)	0	0	1	31	30	1	30 (96.8)	31	0	0
*Klebsiella aerogenes*	30	30	0	0	28 (93.3)	30 (100)	0	0	0	30	30	0	28 (93.3)	30	0	0
*Proteus mirabilis*	30	30	0	0	29 (96.7)	30 (100)	0	0	0	30	30	0	29 (96.7)	30	0	0
*Citrobacter koseri*	30	30	0	0	28 (93.3)	30 (100)	0	0	0	30	30	0	28 (93.3)	30	0	0
*Morganella morganii*	30	30	0	0	30 (100)	30 (100)	0	0	0	30	30	0	30 (100)	30	0	0
*Providencia stuartii*	30	30	0	0	30 (100)	30 (100)	0	0	0	30	30	0	30 (100)	30	0	0
*Proteus vulgaris*	30	30	0	0	29 (96.7)	30 (100)	0	0	0	30	30	0	29 (96.7)	30	0	0
**Clinical Total (%**)	**528**	**516** (**97.7**)	**7** (**1.3**)	**5** (**0.9**)	**504** (**95.5**)	**521** (**98.7**)	**0** (**0**)	**1** (**0.2**)	**6** (**1.1**)	**573**	**560** (**97.7**)	**13** (**2.3**)	**539** (**94.1**)	**571** (**99.7**)	**1** (**7.7**)	**1** (**0.2**)
**Overall Total (%**)	**602**	**577** (**95.8**)	**16** (**2.7**)	**12** (**2.0**)	**576** (**95.7**)	**590** (**98.0**)	**0** (**0**)	**1** (**0.2**)	**11** (**1.8**)	**650**	**621** (**95.5**)	**29** (**4.5**)	**613** (**94.3**)	**643** (**98.9**)	**6** (**20.7**)	**1** (**0.2**)

^*a*^ *K. oxytoca* is not included in the FDA(510k)-clearance for ETEST AZA (12).

^*b*^ AZA, Aztreonam-avibactam; FDA, Food and Drug Administration; EUCAST, European Committee on Antimicrobial Susceptibility Testing; S, Susceptible; I, Intermediate; R, Resistant; VME, Very Major Error; ME, Major error; mE, minor error; EA, Essential agreement; CA, Categorical agreement.

^*c*^ –, not defined.

EUCAST AZA breakpoints for *Enterobacterales* do not include an “intermediate” (susceptible increased exposure) category (14); instead, isolates with MICs ≤4/4 µg/mL are classified as susceptible, whereas those with MICs >4/4 µg/mL are considered resistant (Table 1). Based on these interpretative criteria, 20.8% (16/77) challenge and 2.3% (13/573) clinical isolates tested as resistant to AZA. Among the 77 challenge isolates, CA was 93.5% (72/77), and EA was 96.1% (74/77), with a VME rate of 31.3% observed from five *E. coli* isolates. Clinical isolates demonstrated a 99.7% (571/573) CA and 94.1% (539/573) EA, 7.7% (1/13) VME, and 0.2% (1/560) ME. When combining challenge and clinical isolates for a full-scale analysis (*N* = 650), the combined set achieved an overall CA of 98.9% (643/650), 94.3% (613/650) EA, 0.2% (1/573) MEs, and 20.7% (6/21) VMEs when using EUCAST breakpoints. All VME (6/29) were observed in *E. coli*.

**Fig 1:**
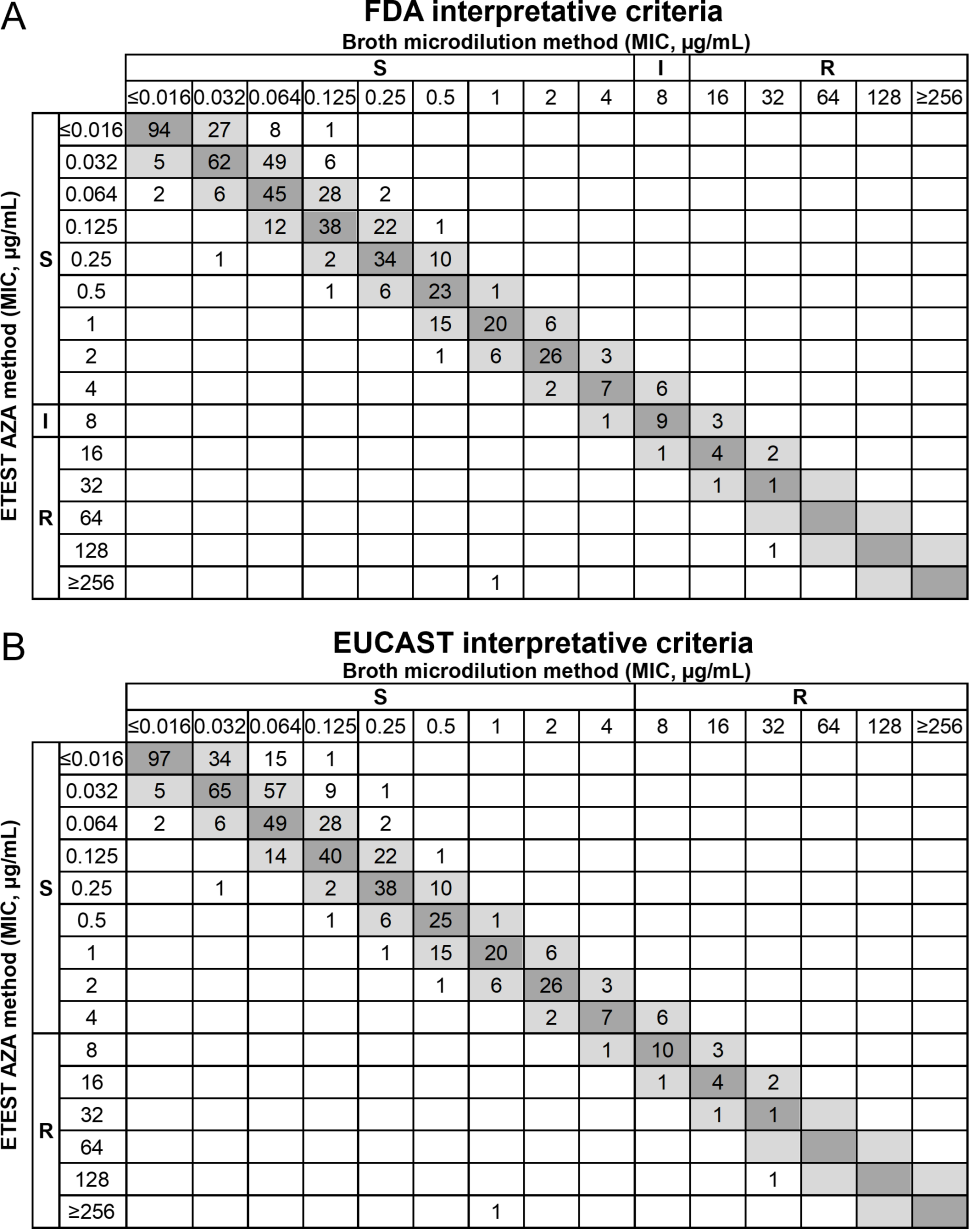
Distribution of MIC values from ETEST AZA and BMD on clinical and challenge isolates. (**A**) FDA breakpoints (*N* = 602) and (**B**) EUCAST breakpoints (*N* = 650), with their respective interpretation noted on the top and left of the plot. Gray rectangles represent MICs in essential agreement, defined as MICs within ±1 doubling dilution. Abbreviations: MIC, minimal inhibitory concentration; AZA, aztreonam-avibactam; BMD, broth microdilution; S, susceptible; I, intermediate; R, resistant.

A total of 508 ETEST AZA MICs were evaluable for trending (Fig. 1). Of these, 53.0% (269/508) showed exact agreement with the reference method, while 34.5% (175/508) isolates tested lower and 12.6% (64/508) tested above the BMD result, resulting in an overall trending of −21.9%. For the following four species, *K. pneumoniae*, *Morganella morganii*, *Proteus vulgaris,* and *Providencia stuartii*, trending is considered significant at more than −30% per the FDA regulatory requirements and is therefore addressed as a note in the US FDA label (13).

Bias per ISO requirements was calculated at −26.5%, based on 10.3% (67/650) of isolates testing higher than the reference method and 36.8% (201/546) isolates testing lower than the reference method. These data indicate that ETEST AZA MICs tended to be in exact agreement or at least one doubling dilution lower compared to the reference BMD method; however, clinical results performance meets the ISO criteria within full-scale bias ≤ ± 30%.

## DISCUSSION

The increasing incidence of MBL-producing *Enterobacterales* has driven the need for novel antimicrobial combinations and corresponding susceptibility testing methods. This multicenter evaluation of ETEST AZA demonstrated high essential and categorical agreement with the reference broth microdilution method across a diverse set of clinical and challenge *Enterobacterales* isolates, supporting its utility for routine clinical microbiology use. One species, *K. oxytoca,* showed suboptimal EA but 100% CA. Notably, all 11/48 *K. oxytoca* isolates with discordant EA results tested with MICs in the lower end of the ETEST AZA concentration range (0.064–0.25 µg/mL). Based on these findings, testing of *K. oxytoca* with ETEST AZA is not included in the final FDA 510(k) clearance (6, 13).

Data on the accuracy of gradient diffusion methods for AZA remain limited as compared to broth microdilution methods. One other gradient diffusion method has been evaluated, the Liofilchem aztreonam-avibactam gradient strip (15). In this single-center study, 103 clinical isolates and 31 resistant *Enterobacterales* isolates from the CDC and FDA Antimicrobial Resistance (AR) Bank were tested. The Liofilchem strip demonstrated an EA of 94.0% (126/134) and a CA of 98.5% (132/134). In contrast to our findings of suboptimal EA performance for *K. oxytoca*, Lemon et al. reported suboptimal performance of the Liofilchem for other species, including CA below 90% for *E. coli* (80.0%) and EA below 90% for the *E. cloacae* complex (83.3%). Of note, Lemon et al. tested only two clinical isolates of *K. oxytoca*. These discrepancies were not linked to specific resistance mechanisms but were postulated to be associated with decreased permeability and/or altered penicillin-binding proteins in *Enterobacterales* isolates (16–19). Mucoid phenotypes were not associated with essential or categorical errors in this study. A total of seven mucoid strains were tested (three *K. pneumoniae*, one *E. coli*, one *C. koseri*, and one *K. oxytoca*). Six of the seven isolates demonstrated essential and categorical agreement using both FDA and EUCAST interpretive breakpoints, with the exception of one *K. pneumoniae* isolate. A future side-by-side comparison of gradient diffusion test strips to the reference BMD method using isolates with well-characterized resistance mechanisms may further elucidate these observed differences.

The observed negative bias in ETEST AZA MICs has possible implications for clinical reporting. Because EUCAST does not define an intermediate interpretive category for AZA, the tendency to underestimate MICs near the susceptibility breakpoint increases the potential for VMEs. Laboratories that use EUCAST interpretive criteria should be aware of this limitation in the WW Package Insert (14). Therefore, confirmatory testing with reference BMD as a comparator method or an alternative testing should be considered only when ETEST AZA indicates susceptibility for isolates with MICs close to the breakpoint. Performance was not assessed using the CLSI interpretative criteria due to unpublished breakpoints in 2025. However, the CLSI approved the same FDA breakpoints after the study concluded, and these interpretive criteria are published in the 36th edition (20).

Our study’s strengths include its multicenter design spanning three geographically diverse regions, the use of gold standard BMD as the reference method, and comparative analysis of both FDA and EUCAST interpretive criteria. This study does have limitations. First, the number of AZA non-susceptible isolates was low (*n* = 29) compared to the total number tested, with about half of these coming from the challenge isolate collection. This limited representation of resistant isolates reflects the recent establishment of interpretive breakpoints and the relatively new clinical adoption of AZA, making it challenging to fully assess performance across all resistance profiles. However, this is the largest study examining gradient diffusion strip testing for AZA to date. Second, testing was conducted using media from a single manufacturer. Prior studies using broth disk elution methods with aztreonam and ceftazidime-avibactam disks have documented variability in results based on the manufacturer, which could potentially impact susceptibility outcomes (4).

In conclusion, ETEST AZA performance met all ISO and FDA regulatory requirements. Our findings support the use of ETEST AZA as an accurate and reproducible method for aztreonam-avibactam susceptibility testing in *Enterobacterales* when applying both the FDA and the EUCAST breakpoints, with the important exception of *K. oxytoca* when the FDA breakpoints are used. Adoption of this gradient diffusion method may help laboratories streamline susceptibility testing for this novel agent, ensuring timely and reliable results to guide appropriate therapy.
